# SRPX2 Promotes Tumor Proliferation and Migration via the FAK Pathway in Papillary Thyroid Carcinoma

**DOI:** 10.1155/2022/5821545

**Published:** 2022-11-03

**Authors:** Ning Luo, Yanfei Tan, Hong Deng, Weiling Wu, Lang Mei, Xinping Huang, Yu Qin, Hongbo Zhu, Chang Liu

**Affiliations:** ^1^Department of Metabolism and Endocrinology, The First People's Hospital of Chenzhou, Chenzhou 423000, China; ^2^Department of Metabolism and Endocrinology, The Affiliated Chenzhou Hospital, Hengyang Medical School, University of South China, Hengyang 421001, China; ^3^Department of Outpatient, Sun Yat-Sen University Cancer Center, State Key Laboratory of Oncology in South China, Collaborative Innovation Center of Cancer Medicine, Guangzhou 510060, China; ^4^Department of Medical Oncology, The First Affiliated Hospital, Hengyang Medical School, University of South China, Hengyang 421001, China

## Abstract

Thyroid cancer is the most common form of endocrine cancer around the world, and among which papillary thyroid carcinoma (PTC) is the most ubiquitous pathological sub-kind. Sushi repeat-containing protein X-linked 2 (SRPX2) was reported to be an independent prognostic factor and significantly overexpressed in advanced PTC patients. However, the biological functions of SRPX2 remain ambiguous in PTC. Here, we explored SRPX2 expression profiles and functions in PTC, finding that SRPX2 expression was remarkably upregulated in PTC tissues and cell lines. Further colony formation, CCK-8, as well as transwell assay, suggested that SRPX2 silencing remarkably dampened PTC growth and migration. Mouse xenograft models were established to find that SRPX2 silence remarkably suppressed PTC proliferation and migration *in vivo*. Following mechanism studies revealed that SRPX2 realized its functions in the PTC process partially through activating the Focal adhesion kinase (FAK) phosphorylation. In conclusion, this study investigated the functions and mechanisms of the SRPX2/FAK pathway in PTC progression. SRPX2 could act as a prospective biologic signature and therapeutic target molecule for PTC.

## 1. Introduction

Thyroid cancer is often encountered among the most commonly found cancers, and the incidence is located in 9th worldwide in 2020 [1]. Globally, thyroid cancer incidence is increasing yearly, especially in high-income regions, which becomes a growing threat to human health [2], and the increase was almost entirely due to papillary thyroid carcinoma (PTC), the most frequent sub-kind [3]. The prognosis of PTC is generally good nowadays. However, cancer recurrence and metastasis may occur and result in a poor prognosis of advanced PTC. Therefore, it is important to assess the molecular parameters of PTC to better predict the clinical course of PTC and plan a treatment strategy.

Sushi repeat-containing protein X-linked 2 (SRPX2), a chondroitin sulfate proteoglycan, is highly expressed in a variety of cancers, including esophageal squamous carcinoma or gastric cancer [4, 5]. It has been reported that SRPX2 promotes the progress of malignant in osteosarcoma by activating YAP1, which is closely associated with drug resistance, malignant phenotypes as well as expansion of cancer stem cells [6]. In advanced PTC, SRPX2 has been found significantly overexpressed and correlated with poorer disease-free survival. Moreover, SRPX2 has the potential to be an independent prognostic biomarker for advanced PTC [7]. However, the biological functions of SRPX2 remain ambiguous in PTC.

We explored SRPX2 expression profiles and functions in PTC tissues and cells, finding that SRPX2 expression was remarkably upregulated. Further experiments suggested that SRPX2 silencing remarkably dampened growth as well as migration in PTC. Following mechanism studies revealed that SRPX2 realized its functions in the PTC process partially through the activation of focal adhesion kinase (FAK) phosphorylation. Our work explored the functions and mechanisms of the SRPX2/FAK pathway in PTC progression. SRPX2 could act as a prospective biologic signature and therapeutic target molecule for PTC.

## 2. Materials and Methods

### 2.1. Tissue Specimens

Thyroid tissues from cancerous (Tumor group) or neighboring noncancerous (normal group) were obtained in surgery from The First People's Hospital of Chenzhou. All fresh tissues were immediately saved in TRIzol (Invitrogen, USA), followed by qRT-PCR assay. The Medical Ethics Committee of The First People's Hospital of Chenzhou approved this study, which was carried out in conformity with the Declaration of Helsinki. Before taking part in the research, all patients furnished their written informed permission forms.

### 2.2. Cell Culture

Human PTC cells (KTC-1, BCPAP, K1, TPC-1, IHH-4, NPA87) and normal thyroid cells (Nthy-ori3-1) were purchased from the American Type Culture Collection (ATCC, USA). Cells were cultured as documented by the manufacturer at the condition of 37°C and 5% CO_2_. Short tandem repeat DNA profiling was adopted to reauthenticate the cells prior to usage.

### 2.3. Cell Transfection

Briefly, si-SRPX2#1 (CCGGATGAAAGCTACAATGAA), si-SRPX2#2 (CGCAGCCGAATCTGCATGGAA), si-SRPX2#3 (CGAGCCTGTATGTGTAGACAT) or the appropriate control si-NC (GeneCopoeia, USA) were transiently transfected into KTC-1 and TPC-1 in 6-well plates (Lipofectamine 3000, Invitrogen). Cells were harvested for further experiments 48 h later.

### 2.4. shRNA Construction and Lentiviral Infection

SRPX2 shRNAs were synthesized by GeneCopoeia. Lentiviruses expressing sh-SRPX2 were produced by Lenti-PacTM HIV Expression Packaging Kit (GeneCopoeia). KTC-1 and TPC-1 were infected with lentiviruses by Lipofectamine 3000. KTC-1-sh-SRPX2 and TPC-1-sh-SRPX2 cells were selected with 2 *μ*g/mL puromycin.

### 2.5. qRT-PCR

Total RNA was obtained by RNA extraction kit (tissue and cells applicable). SYBR Premix Ex Taq kit (Takara, China) was adopted for SRPX2 expression detection. The primer sequences: SRPX2 forward: 5′-GGTGATCGCAGCCGAATCT-3′, reverse: 5′-CAACGGTGGGTCCCAGTAT-3′; GAPDH forward: 5′-GGAGCGAGATCCCTCCAAAAT-3′; reverse: 5′-GGCTGTTGTCATACTTCTCATGG-3′.

### 2.6. CCK-8 Assay

After transfection with sh-SRPX2 or the control vector sh-NC, cells were transferred into plates (96-well plates, 10^3^ each). 48 h later, 10 *μ*l CCK-8 (GlpBio, USA) solution was added. Then, 490 nM absorbance was measured 2 h later.

### 2.7. Colony Formation Assay

After transfection with sh-SRPX2 or the control vector sh-NC, cells were transferred into plates (6-well plates, 10^3^ each). 14 d later, methanol was used to fix the colonies, followed by crystal violet (0.1%) staining. The colonies were counted under microscopy later.

### 2.8. Transwell Assay

After transfection with sh-SRPX2 or the control vector sh-NC, cells were digested 24 hours after transfection and then seeded into upper chambers (10^5^ cells/well). The upper cells were incubated without FBS, and 20% were in lower cells. 48 h later, 0.6% crystal violet was used to stain the upper chamber cells after cold methanol fixing; the stained cells were counted under microscopy later.

### 2.9. Mouse Xenograft Model

Ethical approval was obtained from the Institute Research Ethics Committee of The First People's Hospital of Chenzhou, and animal experiments were conducted following the institutional standard guidelines from The First People's Hospital of Chenzhou. 2 × 10^6^ cells/mL TPC-1-sh-SRPX2 or TPC-1-sh-NC cells were injected into C57BL/6 nude mice (dorsal flanks, five mice/group). Then tumors were extracted for weight and volume measurement 4 weeks later. 10^5^ TPC-1-sh-SRPX2 or TPC-1-sh-NC cells were inoculated through the tail vein into nude mice (five mice/group) to construct a lung metastasis model. After implantation for 8 weeks. The lungs were obtained followed by a pathology assessment. And the number of macroscopically evident lung metastatic nodules was enumerated and verified via microscopy of HE-stained sections.

### 2.10. Western Blotting

Total PTC cell proteins were extracted by PMSF as well as RIPA lysis, followed by proteins separation, transfer, and primary antibody incubation including FAK (1 : 1000, #AF6397, Affinity, USA), p-FAK (1 : 1000, #AF3398, Affinity), and GAPDH (1 : 1000, #AF7021, Affinity), and secondary antibodies (1 : 3000, #7074S, CST, USA). The target protein was visualized and quantified at last (ECL New England Biolabs, USA).

### 2.11. Immunohistochemistry (IHC) Staining

Formalin-fixed, as well as paraffin-embedded tumor tissues, were sliced into 4 mm sections, followed by deparaffinized, rehydrated, antigen retrieval as well as endogenous peroxidase activity destruction. Next, p-FAK (1 : 100, #AF3398, Affinity), peroxidase-conjugated secondary antibody, DAB solution was for the following *H* & *E* detection.

### 2.12. Statistical Analysis

GraphPad Prism 8.0 software was implemented for data analyses. *T*-tests, as well as one-way variance analysis, were employed in data analysis. All data is given in the form of mean ± SD. When *P* < 0.05, statistical significance is established.

## 3. Results

### 3.1. SRPX2 is Elevated in PTC

**Figure 1:**
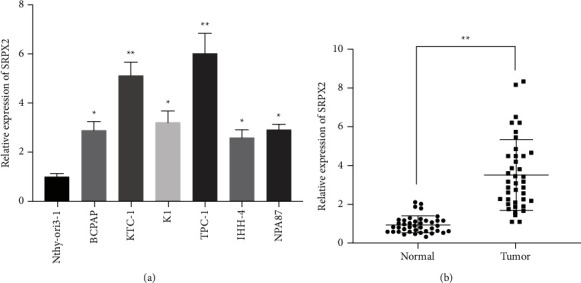
SRPX2 is elevated in PTC. (a) SRPX2 expression in PTC cells. (b) SRPX2 expression in 40 matched PTC tissues and its control tissues. ^*∗∗*^*P* < 0.01, ^*∗*^*P* < 0.05.

SRPX2 participates in the progress of multiple cancers. However, the functions and potential mechanisms of SRPX2 in PTC remain unknown. Studies revealed that SRPX2 is overexpressed in advanced PTC patients and correlated with poorer disease-free survival [7]. Here, qRT-PCR was adopted for the assessment of SRPX2 expression in PTC cell lines as well as tissues. As shown in Figure 1(a), in comparison to the nonmalignant thyroid cell line Nthy-ori3-1, SRPX2 was elevated in PTC cells, particularly in TPC-1 as well as KTC-1. We further verified SRPX2 expression in 40 paired neighboring noncancerous thyroid tissues and PTC tissues. And the data illustrated that, compared to the surrounding nonmalignant thyroid tissues, SRPX2 was remarkably elevated in PTC tissues (Figure 1(b)).

### 3.2. SRPX2 Inhibition Diminished PTC Cell Proliferation as well as Invasion

**Figure 2:**
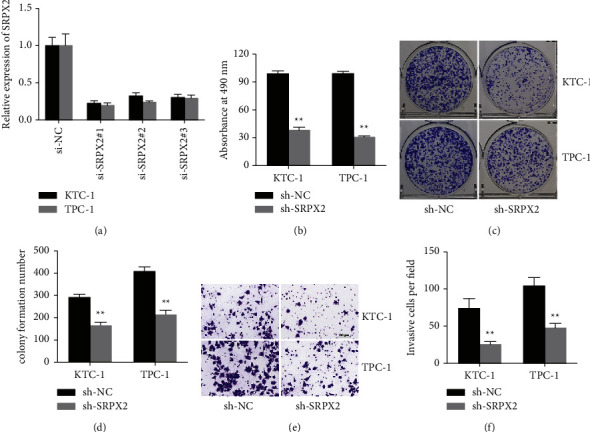
SRPX2 inhibition diminished PTC cell proliferation as well as invasion. (a) The knockdown efficacy of si-RNAs was detected in the KTC-1 as well as TPC-1 cell line. (b) CCK-8 analysis to assess the cell proliferation ability after knockdown of SRPX2 expression. (c) sh-SRPX2 transfected cells were used to assess the cell proliferation ability. (d) Statistic graph of colonies. (e) Transwell images of KTC-1 as well as TPC-1 cell line. (f) The number of infiltrative cells was quantified by ImageJ ^*∗∗*^*P* < 0.01.

Since SRPX2 was upregulated in PTC, we designed si-RNAs to silence SRPX2 and explore its function. As shown in Figure 2(a), SRPX2 expression was remarkably reduced in cells transfected with si-SRPX2#1, which was built into shRNA. CCK-8 proliferation assays illustrated that SRPX2 silencing dampened the proliferation potential of PTC cells (Figure 2(b)). Besides, the colony-forming ability of PTC cells was evidently suppressed when SRPX2 was knocked down (Figures 2(c) and 2(d)). Moreover, transwell assays revealed that SRPX2 silencing expression diminished the invasion ability of PTC cells (Figures 2(e) and 2(f)).

### 3.3. SRPX2 Inhibition Suppressed PTC Proliferation and Migration *in Vivo*

**Figure 3:**
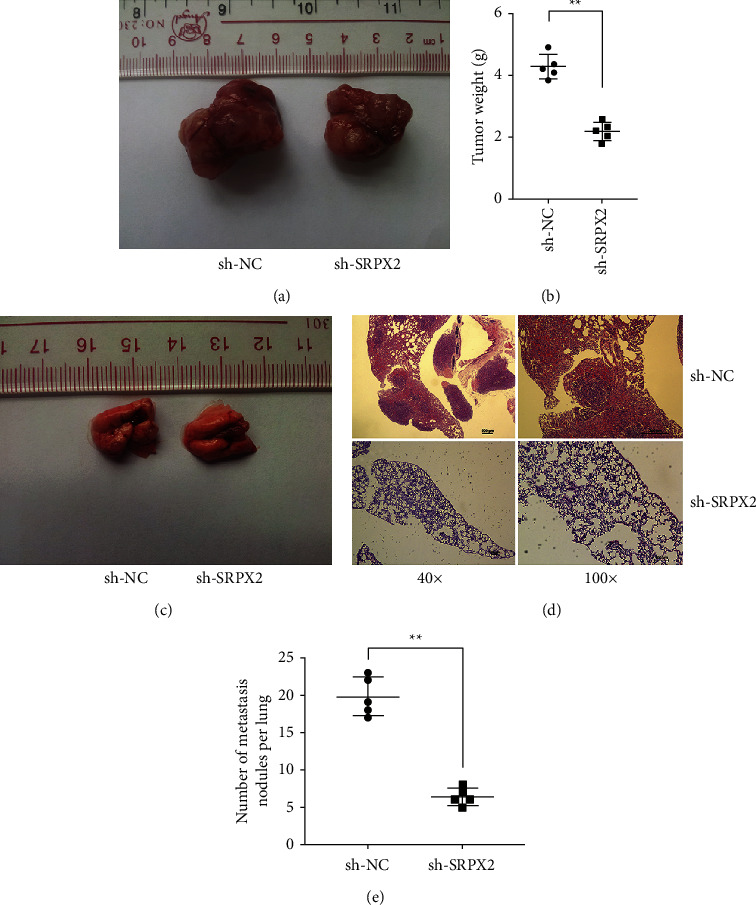
SRPX2 inhibition suppressed PTC proliferation and migration *in vivo*. (a) Images of tumors from different groups. (b) Statistic graph of tumor weights. (c) Images of lung metastatic nodules. (d) Images of HE-stained nodules. (e) Statistic graph of metastatic nodule number. ^*∗∗*^*P* < 0.01.

Next, we continued to investigate the *in vivo* functions of SRPX2. And results manifested that inhibition of SRPX2 expression significantly suppressed tumor growth (Figures 3(a) and 3(b)). Furthermore, the knockdown of SRPX2 expression significantly decreased PTC lung metastasis (Figures 3(c)–3(e)), indicating that SRPX2 silencing curbed PTC proliferation as well as migration *in vivo*.

### 3.4. SRPX2 Inhibition Led to Decreased Phosphorylation Levels of FAK

**Figure 4:**
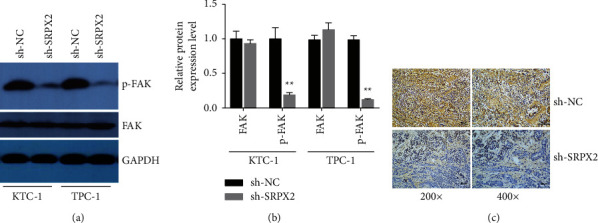
SRPX2 inhibition suppressed PTC proliferation and migration in vivo. (a) KTC-1 and TPC-1 cells transfected with sh-NC or sh-SRPX2 were detected in FAK and p-FAK expression. (b) FAK and p-FAK expression in western blotting assay were quantified by ImageJ software. (c) Representative images of IHC-stainedp-FAK in mouse xenograft tumor tissues. ^*∗∗*^*P* < 0.01.

Protein tyrosine kinase FAK is involved in multiple cancer processes. It has been reported that SRPX2 realizes its functions partly via the FAK-dependent pathway. Therefore, we continued to explore if SRPX2 regulated FAK activating process in PTC cells. The results revealed that p-FAK was strikingly reduced when SRPX2 was silenced in PTC cells (Figures 4(a) and 4(b)). Next, to explore the regulation of SRPX2 in the activation of FAK in PTC *in vivo*, IHC staining was performed in the above mouse xenograft tumor tissues. p-FAK expression was noticeably decreased after the knockdown of SRPX2 (Figure 4(c)). These results indicated that SRPX2 might function partly in a FAK-dependent pathway in PTC progression.

## 4. Discussion

Thyroid cancer has been reported as the most pervasive endocrine cancer in the world, and the incidence keeps increasing for the past decades [8]. And PTC is a type of high-frequency pathological subkind [9]. Although improved systemic therapies, including radioiodine and targeted drugs, are available for PTC, advanced PTC prognosis is still poor [10]. Therefore, it is quite necessary to develop biomarkers and targets that can help better treat PTC.

Anomalous SRPX2 exerts substantial functions in multiple cancers. SRPX2 is a component in an extracellular matrix which involved in tumor formation including colorectal cancer [11], gastrointestinal cancer [12], and prostate cancer [13]. Additionally, SRPX2 exerts essential roles in pan-cancers because of participating in stem cell differentiation of human embryonic [14, 15]. In PTC, SRPX2 has also been found significantly overexpressed and correlated with poorer disease-free survival of advanced PTC [7]. However, the biological functions of SRPX2 in PTC have not yet been reported.

Here, we confirmed SRPX2 expression in PTC cells as well as tissues, manifesting SRPX2 was boosted in PTC (Figure 1). Subsequent functional experiments evinced that SRPX2 silencing dampened the proliferation as well as migration potential in PTC (Figures 2 and 3). All these findings revealed the important role SRPX2 play in PTC. Hence, SRPX2 had the potential to be a treatment target and clinical signature for PTC.

Next, we continued exploring the mechanism involving SRPX2 in PTC progression. FAK belongs to the protein tyrosine kinase families, participating in the movement, progress, self-renewal, and gene expression of cells [16]. Increasing evidence has shown that targeting FAK may be a promising therapeutic strategy for multiple cancers [17]. It has been reported that SRPX2 realizes its functions partly by FAK-dependent pathway. In pancreatic ductal adenocarcinoma, SRPX2 is boosted and contributes to malignant processes through the FAK-dependent pathway [18]. Overexpression of SRPX2 boosts tumor progress by FAK/SRC/ERK pathway in lung cancer [19]. SRPX2 expedites hepatocellular carcinoma by targeting the FAK/AKT pathway and regulating the expression of MMP2/9 [20]. However, the relationship between SRPX2 and FAK in PTC has not yet been reported. Here, we revealed that SRPX2 silencing remarkably decreased p-FAK levels in PTC cells and mouse xenograft tumor tissues (Figure 4), indicating that SRPX2 might function partly in a FAK-dependent pathway to accelerate PTC progression.

## 5. Conclusions

SRPX2 expression was remarkably boosted in PTC. SRPX2 silencing suppressed PTC cell proliferation as well as migration partially via the reduction of FAK phosphorylation. Though the detailed mechanism needs further clarification, this research disclosed the crucial biological functions of SRPX2 in PTC progress. SRPX2 could act as a prospective biologic signature as well as a therapeutic target molecule.

## Data Availability

The data used to support the findings of this study are available from the corresponding author upon request. E-mail could be sent to the address 835478729@qq.com.
